# Performance of Large Language Models and Top-Decile Doctors on an Undergraduate Ophthalmology Examination

**DOI:** 10.7759/cureus.97687

**Published:** 2025-11-24

**Authors:** Oluwaseun Akinniranye, Olusegun Akinniranye

**Affiliations:** 1 Ophthalmology, Princess Alexandra Hospital, Harlow, GBR; 2 Anaesthesiology, Princess Alexandra Hospital, Harlow, GBR

**Keywords:** artificial intelligence and education, duke elder, large language models, multiple-choice questions, ophthalmology

## Abstract

Introduction: The rapid advancements of artificial intelligence (AI) and large language models (LLMs) have led to their increasing use in medical education and clinical practice. While studies demonstrate their proficiency in passing medical examinations, it remains unclear how their performance compares to that of human experts on a challenging, domain-specific examination.

Methods: This study compared the performance of four contemporary LLMs (ChatGPT-5 (OpenAI, San Francisco, CA), Claude Sonnet 4 (Anthropic, San Francisco, CA), Gemini Pro (Google, Mountain View, CA), and Perplexity (Perplexity AI, San Francisco, CA)) with a cohort of three doctors who had all recently scored in the top 10% of candidates on the Duke Elder Undergraduate Prize Examination. Participants were assessed on a 100 multiple-choice question (MCQ) examination that mimicked the style and difficulty of the prize exam. A two-tailed independent samples t-test was used for a primary comparison of the pooled LLM and pooled doctor cohorts, while a one-way ANOVA with Tukey's HSD post-hoc analysis was used for a secondary comparison of the four individual LLMs and the pooled doctor cohort.

Results: The pooled LLM cohort, with a mean score of 85.50%, performed similarly to the pooled doctor cohort, which scored 75.67%. A primary analysis revealed no statistically significant difference between these groups (p = 0.0533). However, a secondary analysis found a significant difference among the individual LLMs and pooled doctor cohorts (p = 0.0370), with a post-hoc analysis showing that Claude Sonnet 4 (91.00%) significantly outperformed the pooled doctors (p = 0.0268). A sub-analysis on the optics subsection of the exam showed no significant difference between the pooled LLM cohort and the pooled doctor cohort (p = 0.5072, and no significant difference between individual LLMs and the pooled doctor cohort (p = 0.3545, respectively).

Conclusion: The findings suggest that popular LLMs, particularly Claude Sonnet 4, have achieved a level of performance on par with high-performing doctors for a challenging ophthalmology examination. This study highlights the immense potential of LLMs as valuable educational tools in ophthalmology, while also underscoring the importance of their continued evaluation against rigorous clinical benchmarks and human oversight in practice.

## Introduction

LLMs' efficacy in passing medical examinations

The rapid advancement of large language models (LLMs) is transforming various fields, including medicine, where their efficacy in passing medical examinations has shown a significant increase. LLMs such as ChatGPT (Open AI, San Francisco, CA) have demonstrated improved performance with each new iteration, with recent studies showing that ChatGPT 4.0 outscored its predecessor, ChatGPT 3.5, on major exams like the United States Medical Licensing Examination (USMLE) [1], the Membership of the Royal College of Surgeons (MRCS) examination [2], and the Fellowship of the Royal College of Ophthalmologists (FRCOphth) exams [3]. This continuous improvement is attributed to their training on vast datasets, allowing them to approach expert-level clinical knowledge and reasoning, particularly in specialized fields like ophthalmology [4].

The Duke Elder Undergraduate Prize

The Duke Elder Undergraduate Prize is a prestigious and high-stakes examination administered by the Royal College of Ophthalmologists. It is designed for medical students who have completed their ophthalmology curriculum, with a high standard that is substantially more challenging than typical undergraduate exams. Candidates who score in the top 10% are awarded two points toward their national ophthalmology training application, while those in the top 60% receive 0.5 points. A substantial and conceptually distinct component of this exam is the optics section, which requires the application of optical laws in a clinical context. We hypothesized that this unique and potentially less common subject matter would pose a particular challenge for LLMs due to a theoretical lack of specific training data.

Advancement of AI

Recently, LLMs have been compared to physicians in their ability to complete medical examinations. A significant body of research demonstrates LLMs' growing proficiency, with one study showing that specialist physicians rated LLM-generated examination answers as equally safe as those from generalist physicians [5]. Another study from Oxford highlighted the superior diagnostic capabilities of LLMs, which correctly identified conditions with 94.9% accuracy, in stark contrast to humans using LLMs, who achieved less than 35% accuracy [6].

The rapid and continuous advancement of AI necessitates ongoing evaluation of its capabilities, particularly in high-stakes fields like medicine. As described above, multiple studies have already demonstrated LLMs' ability to pass medical exams, but it remains unclear how their performance compares to that of human experts on a challenging, domain-specific examination. With the recent release of ChatGPT-5 in August 2025, this study aims to evaluate the performance of four contemporary large language models (LLMs) on an ophthalmology-related medical exam. In this study, ChatGPT-5 by OpenAI, Claude Sonnet 4 (Anthropic, San Francisco, CA), Gemini Pro (Google, Mountain View, CA), and Perplexity (Perplexity AI, San Francisco, CA) are compared on their performance against each other. We will also compare their accuracy against a benchmark of human expertise represented by three doctors who all scored in the top 10% on the Duke Elder exam within the preceding year. This cross-sectional evaluation is crucial for understanding how these rapidly evolving models perform against human experts on a domain-specific and challenging assessment.

Aims

The primary aim of this study was to evaluate and compare the overall accuracy of a cohort of LLMs with that of a human cohort consisting of doctors who had achieved a top-decile score on the Duke Elder examination. In addition, a planned sub-analysis focused specifically on the optics section of the examination, allowing for a direct comparison of performance between the LLM cohort and the human cohort in this distinct and often challenging domain.

## Materials and methods

Question bank

The examination used in this study was a 100-item, single-best-answer, MCQ exam from the ‘300 MCQs for the Duke Elder Ophthalmology Exam’ by Patel et al. [7]. As official past papers for the Duke Elder examination are not publicly released, a Duke Elder-style exam was chosen to serve as a high-fidelity benchmark. To mitigate the risk of data contamination from LLM pre-training sources, the exam was sourced from a textbook not freely available on the internet. The textbook was selected after a subjective assessment by the participating doctors as being the most difficult of five shortlisted Duke Elder-style exams from three different sources. For the purpose of sub-analysis, each question was classified according to one of the ten sub-speciality areas defined by the Royal College of Ophthalmologists' exam blueprint.

Cohorts

Four LLMs were included in this study: ChatGPT-5 by OpenAI, Claude Sonnet 4 by Anthropic, Gemini Pro by Google, and Perplexity by Perplexity AI.

The human benchmark was represented by three doctors who all scored in the top 10% on the Duke Elder examination within the preceding year. As the exam has no fixed pass mark, this cohort provided a robust, top-decile benchmark of human expertise.

Procedures

To mimic the conditions of a real-world exam, the doctors were given 133 minutes to complete the 100-question exam, approximating the Duke Elder's rate of 1.33 minutes per question. The correct answer for each question was taken from the back of the textbook. Each correct answer was given a value of 1, and each incorrect answer was given a value of 0 to allow for quantitative statistical analysis.

All 100 questions from the exam were manually input into each LLM from August 19 to 26, 2025. Questions were not paraphrased. The initial prompt provided to each LLM was: 'I will be asking you a set of 100 multiple-choice ophthalmology-based questions. Please answer by picking the letter which is most correct'. The LLMs were not given access to any external tools or browsing capabilities. Similar to the doctors, the LLMs were given 1.33 minutes per question. If the LLM failed to answer a question within 1.33 minutes, the answer was deemed to be incorrect.

Statistical analysis

Our primary analysis was a two-cohort comparison of the pooled LLM cohort versus the pooled doctor cohort. For this, an independent samples t-test was performed to compare their mean scores on both the total exam and the optics subsection.

The secondary analysis was a comparison of five cohorts: the four individual LLMs and the pooled doctor cohort. A one-way ANOVA was used to compare the mean scores on both the total exam and the optics subsection. If the ANOVA showed a significant difference, a Tukey's HSD post hoc analysis was performed to determine which specific cohorts differed from each other.

The study was designed with a statistical power of 80% to detect a 10% difference in overall accuracy between the LLM and doctor cohorts, using a two-sided alpha level of 0.05. To achieve this, we performed a power analysis using the observed cohorts’ means from the study: 85.5% for the pooled LLMs and 75.7% for the pooled doctors, an absolute difference of 9.8%. Given our sample size of 100 questions and the study's design, which accounts for the clustering of responses by question, we applied a design-effect adjustment. This approach used a conservative estimate for the item-level intra-class correlation coefficient (ICC) of approximately 0.15, which accounts for the similarity of responses to a single question. Our calculations indicated that the study had an estimated power of 0.80, confirming that our 100-item exam was adequately powered to detect the observed a 9.8% difference under plausible assumptions. Sensitivity analyses further supported this conclusion, showing that the power remained high at 0.83 with a lower intra-class correlation coefficient of 0.10 and was 0.76 with a higher intra-class correlation coefficient of 0.20.

## Results

Overall exam results

Table 1 shows the number of correct answers per sub-speciality for each participant, alongside the total questions.

**Table 1:** Total number of correct answers from seven participants (four LLMs and three doctors) for each sub-speciality section of a Duke Elder-style examination. LLM = Large language model

Sub-speciality	ChatGPT-5	Gemini Pro	Claude Sonnet 4	Perplexity	Doctor 1	Doctor 2	Doctor 3	Total number of questions
Cornea and external eye disease	11	11	12	11	9	10	10	12
Cataract and the lens	4	4	4	4	4	4	4	5
Glaucoma	4	4	4	3	3	3	3	4
Medical retina and vitreo-retinal surgery	9	9	9	8	7	8	7	9
Strabismus and paediatric ophthalmology	4	4	4	5	4	4	3	5
Neuro-ophthalmology	6	6	7	6	5	5	4	8
Ocular adnexa and orbital disease	5	3	5	5	3	3	2	5
Refractive errors and optics	18	22	24	22	20	23	20	27
Anatomy and embryology	17	19	20	19	17	15	19	21
Uveitis	2	4	2	2	2	3	3	4

Table 2 presents the overall score for each of the seven participants on the exam, alongside the averages for the LLMs and the doctors.

**Table 2:** Overall score from seven respondents (four LLMs and three doctors) for a Duke Elder-style examination, with averages for the LLM and doctor cohort included. LLM = Large language model

ChatGPT-5 (%)	Gemini Pro (%)	Claude Sonnet 4 (%)	Perplexity (%)	LLM average (%)	Doctor 1 (%)	Doctor 2 (%)	Doctor 3 (%)	Doctor average (%)
80.00	86.00	91.00	85.00	85.50 ± 4.51	74.00	78.00	75.00	75.67 ± 2.08

**Figure 1:**
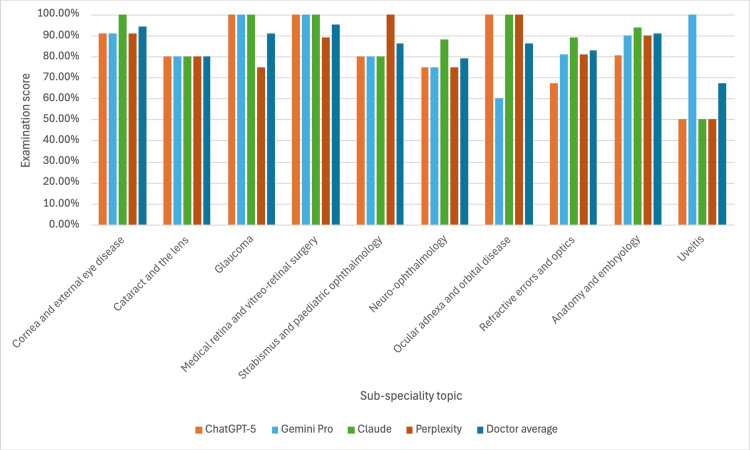
The scores of four LLMs and a pooled cohort doctors in the 10 sub-specialities of a Duke Elder-style examination The five cohorts in this study included four LLMs (ChatGPT-5, Gemini Pro, Claude Sonnet 4, and Perplexity) and a pooled cohort of doctors (n = 3). The doctors had all previously scored in the top 10% of candidates on the Duke Elder examination within the preceding year. Visually, the five cohorts performed similarly across all 10 sub-specialities. Among the cohorts, Claude Sonnet 4 demonstrated the highest overall performance, scoring the highest or joint-highest in eight of the 10 sub-specialities. LLM = Large language model

Figure 1 details the scores of the three LLMs and the average scores of the three doctors for the 10 sub-specialities of the Duke Elder-style exam.

Pooled LLMs vs pooled doctors

The following results compare the average overall and optics scores for the pooled LLM cohort against the pooled doctor cohort.

Overall Examination Score

**Figure 2:**
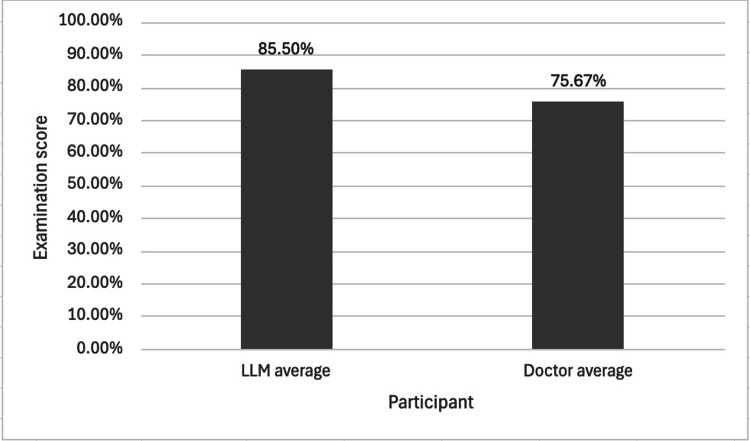
The mean scores of a pooled cohort of LLMs and a pooled cohort of doctors in a Duke Elder-style examination. For the pooled LLM cohort, the sample size was n=4 (ChatGPT-5, Gemini Pro, Claude Sonnet 4, and Perplexity), and for the pooled doctor cohort, n=3. All participating doctors had previously scored within the top 10% of candidates on the Duke Elder examination within the preceding year. Although the LLM cohort showed a nearly 10% increase in mean exam score, a two-tailed independent samples t-test revealed no statistically significant difference between the two cohorts (p = 0.0533). This finding suggests that this cohort of LLMs and this cohort of doctors had no significant difference in performance. LLM = Large language model

Figure 2 shows the mean examination score of the pooled LLMs and the pooled doctors. 

Optics Sub-speciality Score

**Figure 3:**
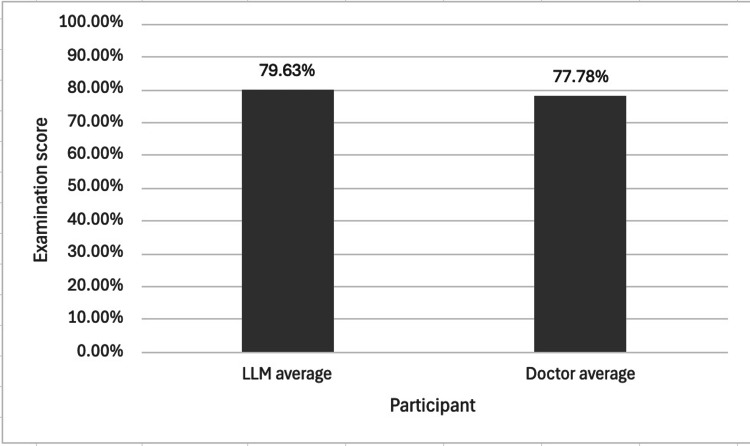
The mean scores of a pooled cohort of LLMs and a pooled cohort of doctors in the optics sub-speciality section of a Duke Elder-style examination. For the pooled LLM cohort, the sample size was n=4 (ChatGPT-5, Gemini Pro, Claude Sonnet 4, and Perplexity), and for the pooled doctor cohort, n=3. All participating doctors had previously scored within the top 10% of candidates on the Duke Elder examination within the preceding year. The LLM cohort showed nearly a 2% increase in mean optics score. A two-tailed independent samples t-test showed no statistically significant difference between the two cohorts (p = 0.5072). This finding suggests that this cohort of LLMs and the cohort of doctors in this study showed no significant difference in performance on optics. LLM = Large language model

Figure 3 shows the mean optics score of the pooled LLMs and the pooled doctors. 

Separate LLMs vs pooled doctors

The following results compare the average overall and optics scores for the individual LLMs against the pooled doctor cohort.

Overall Examination Score

**Figure 4:**
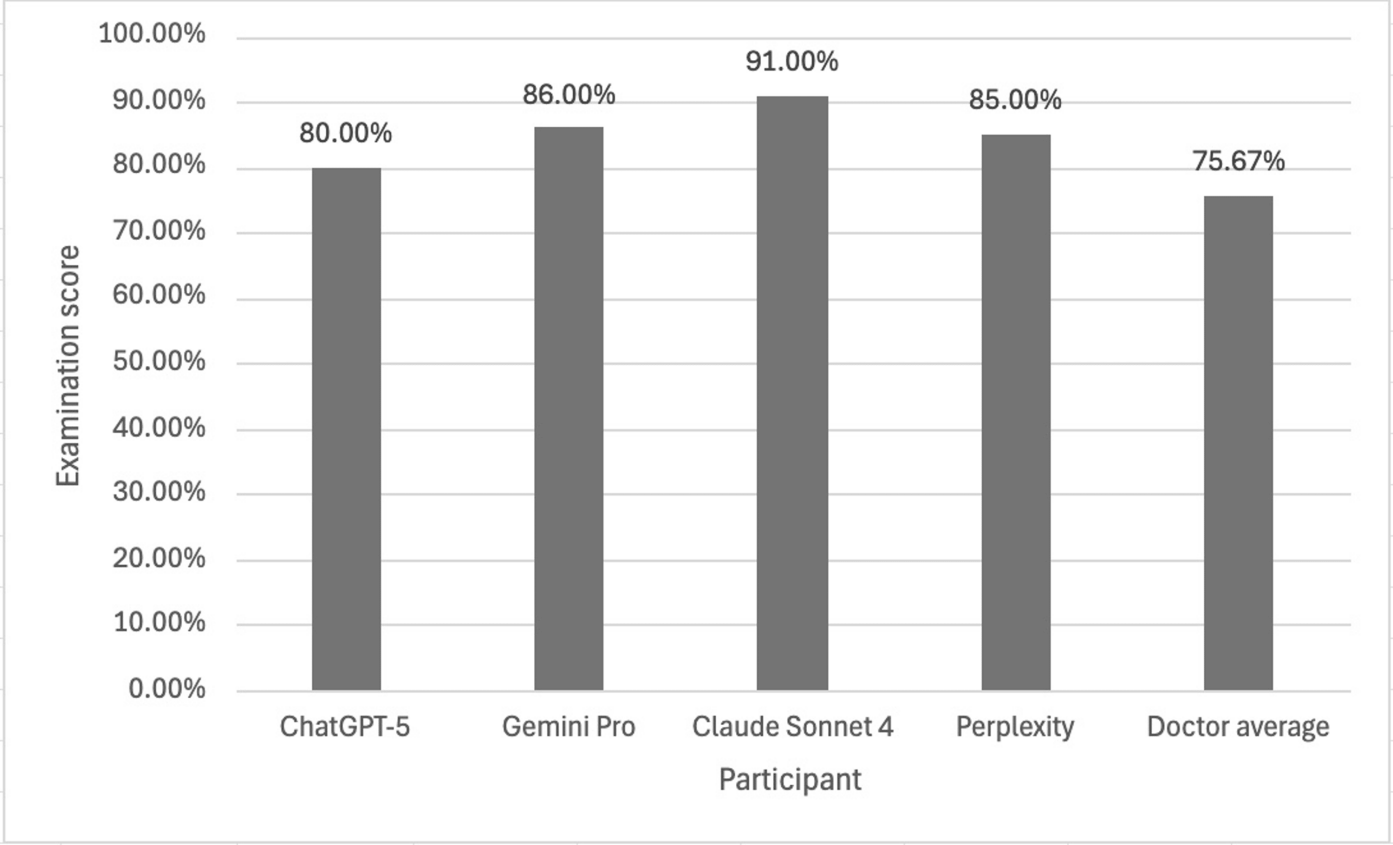
The mean scores of different LLMs and a pooled cohort of doctors in a Duke Elder-style examination For the pooled doctor cohort, the sample size was n = 3, and all participating doctors had previously scored within the top 10% of candidates on the Duke Elder examination within the preceding year. A one-way ANOVA revealed a statistically significant difference in the mean examination scores across the cohorts at a 95% confidence interval (p = 0.0370). A Tukey’s HSD post-hoc analysis confirmed a significant difference between the Claude Sonnet 4 and doctor cohorts (p = 0.0268), with mean scores of 91.00% and 75.67% respectively. No other statistically significant differences were observed between any of the other cohorts. These findings suggest that Claude Sonnet 4 achieved a significantly higher exam score than the doctors in this study, while the other LLMs showed no significant difference to both the doctor cohort and each other. LLM = Large language model

Figure 4 shows the mean examination score of the LLMs and the pooled doctors.

Optics Sub-speciality Score

**Figure 5:**
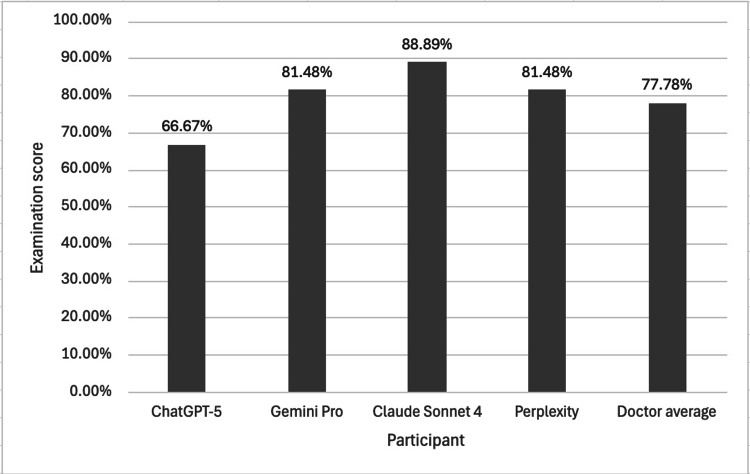
The mean scores of different LLMs and a pooled cohort of doctors in the optics sub-speciality section of a Duke Elder-style examination For the pooled doctor cohort, the sample size was n = 3, and all participating doctors had previously scored within the top 10% of candidates on the Duke Elder examination within the preceding year. Although Claude Sonnet 4 was the highest performer and scored over 7% higher than the next best score, a one-way ANOVA revealed no significant difference between the cohorts’ scores (p = 0.3545). This suggests that these LLMs and this cohort of doctors showed no significant difference in scores. LLM = Large language model

Figure 5 shows the mean optics score of the LLMs against the doctors.

Summary of key findings

The visual data (Figure 1) showed that Claude Sonnet 4 was the highest-performing participant, scoring the highest or joint-highest in eight of the ten sub-specialities 

The overall mean exam score for the pooled LLMs cohort was 85.50%, which was nearly 10% higher than the 75.67% mean score of the pooled doctor cohort. However, a two-tailed independent samples t-test revealed no statistically significant difference between the two groups (Figure 2, p = 0.0533). 

When the five cohorts were analysed (four LLMs and one pooled cohort of doctors), a one-way ANOVA showed a statistically significant difference in mean examination scores (Figure 4, p = 0.0370). A subsequent Tukey’s HSD post-hoc analysis confirmed a significant difference only when comparing Claude Sonnet 4 to the doctor cohort (Figure 4, p = 0.0268). Claude Sonnet 4 scored 91.00% compared to the doctors' average of 75.67%. No other statistically significant differences were observed between any of the other cohorts. 

For the optics sub-speciality, the pooled LLM cohort demonstrated a higher mean score than the pooled doctor cohort, yet a two-tailed independent samples t-test revealed no statistically significant difference between the two groups (Figure 3, p = 0.5072). Similarly, a one-way ANOVA across the five cohorts showed no significant difference in scores (Figure 5, p = 0.3545). These findings show that although the LLMs achieved higher mean scores in the optics section, this difference was not statistically significant compared to the doctors’ performance in this study. 

## Discussion

Overall exam results 

The primary analysis of this study found no statistically significant difference in overall exam scores between the pooled LLM cohort and the pooled doctor cohort (Figure 2, p = 0.0533). 

This finding suggests that, collectively, the LLMs showed no difference in performance to high-achieving doctors on a challenging, ophthalmology-specific examination. This result aligns with previous research, which has demonstrated that LLMs like ChatGPT-4 show no significant difference in performance to expert ophthalmologists on MCQ-based postgraduate exams [4]. The significant advancement in LLM performance is further highlighted by multiple studies showing that ChatGPT-4 significantly outperforms its predecessor, ChatGPT-3.5, on ophthalmological board examinations [3,4,8]. For example, one study noted that Chat GPT-4 scored 95.54% compared to a cohort of medical students' 72.15% and achieved 100% accuracy on another set of questions versus Chat GPT-3.5's 64% [9]. 

The exponential growth in LLM performance, particularly the massive increase in training data from ChatGPT-3.5 to ChatGPT-4, set a high bar for newer models. While it was expected that models like ChatGPT-5 would similarly outperform their predecessors, our findings suggest a more nuanced picture. The lack of a statistically significant difference between the pooled LLM and doctor cohorts (Figure 2, p = 0.0533) may indicate a knowledge plateau for this type of exam. 

However, this finding is complicated by the significant difference revealed in our secondary analysis, with Claude Sonnet 4 outperforming the doctor cohort (Figure 4, p = 0.0268). This suggests that the training data and nuances of each LLM can play a critical role in their knowledge base. Claude’s superior performance (Figures 1, 4, 5) could be due to its greater reasoning architecture, fine-tuning for ophthalmology medical contexts, or simply reflects the varied performance of LLMs across clinical scenarios. Other studies offer an alternative conclusion to this study’s results. Studies have shown that GPT-4o achieved higher diagnostic accuracy than Claude Sonnet 3.5 on a 20-case corneal disease series [10], and GPT-4o outperformed Claude Sonnet 3.5 (56.29% vs ~40.03%) in retinal disease diagnosis [11]. This underlines how a model's relative performance can be task-dependent, and for ophthalmologists, context will be highly important in deciding which LLM may best aid clinical knowledge. 

Optics sub-speciality score 

This study’s sub-analysis on the optics section did not support our initial hypothesis that LLMs would find this sub-speciality particularly challenging. Both the pooled LLM against doctor cohort comparison (Figure 3, p = 0.5072) and the individual LLM against doctor cohort comparison (Figure 5, p = 0.3545) revealed no statistically significant difference in scores, suggesting no difference in the performance of LLMs and doctors. 

This is a crucial finding, as optics tests the application of principles to a clinical context, which requires a deeper understanding than the simple factual recall that is often found in other sub-specialties for the Duke Elder exam. The proficiency demonstrated by LLMs in this technically challenging domain highlights their potential to move beyond factual memorization into more complex, application-based reasoning. This proficiency underscores how LLMs can serve as a valuable educational tool for doctors and medical students, aiding in both knowledge-based learning and the application of complex rules to clinical scenarios. 

Drawbacks and ethical considerations 

A balanced discussion of LLMs in medicine must address their drawbacks and potential risks. While they show impressive knowledge, they may also provide incorrect clinical information with high confidence, which can be particularly dangerous in a clinical setting. A study demonstrated that when testing LLMs on clinical MCQs, worse-performing models demonstrated higher confidence in their answers [12]. This could make them poor teaching resources, as they might reinforce incorrect knowledge without the ability to reason or admit uncertainty. 

Furthermore, a significant concern is the potential for cognitive deskilling. This is highlighted by research articles, which suggest that over-reliance on AI tools can reduce opportunities for clinicians to practice diagnostic reasoning and erode medical expertise [13]. This reliance could also lead to a shallow grasp of medical topics, as LLMs may prioritize factual recall over true understanding and reasoning. 

The ethical implications of AI in medical education are also a major concern. The use of vast datasets to train LLMs raises questions about data privacy and the potential for patient information breaches. In addition, if the training data is not representative of diverse patient populations, the LLMs may perpetuate and amplify systemic biases, leading to disparate outcomes in healthcare. This has been demonstrated in radiology, where AI models underdiagnosed findings in marginalized groups, particularly Black female patients [14]. 

Limitations 

This study has several limitations that should be considered when interpreting its findings. The primary limitation is that our analysis was based on a single 100-question exam, which may not be fully representative of all Duke Elder-style examinations. This small dataset also limits the generalisability of our findings. A better assessment would involve using a wider range of questions from multiple exams or, ideally, real-life clinical scenarios to better simulate the complexities of medical practice. 

A further limitation relates to reproducibility. Because both the doctors and the LLMs would likely retain exposure to the questions after the initial assessment, repeating the exam under identical conditions could introduce learning or memorization effects, leading to inflated performance and distorted comparisons. Therefore, any attempt to replicate this study would require the creation of a new question set of similar scope and difficulty, which may not be identical to the original exam. This limits the ease of direct reproducibility.

Furthermore, our methodology, which graded only correct or incorrect answers, did not account for the quality of the participants' reasoning. A clinician's ability to reason through a problem may be a better indicator of competence than simply arriving at the right answer. This study could be improved by recording and analysing the reasoning provided by doctors and LLMs. The reliance on multiple-choice questions is a known limitation in LLM research, as this format does not fully capture the nuanced skills of clinical performance, patient care, and communication. 

There are also several limitations specific to the use of LLMs. Despite our best efforts to use an exam from a non-public source, we cannot guarantee that some of the questions were not included in the LLMs' training datasets, which introduces the risk of dataset contamination. Furthermore, as the training data used for LLMs is not publicly disclosed, it is not possible to determine precisely what ophthalmic material they were trained on, nor to directly compare these sources with the doctors’ knowledge base. The doctors’ understanding was acquired over several years through varied clinical training environments, teaching styles, and textbooks, making their educational exposure inherently diverse and difficult to standardize. The rapid progression of LLMs also means that the results from this study may quickly become outdated as newer, more capable models are released. Finally, LLMs are known to be inconsistent, sometimes giving different answers to the same question when retested. One study has noted LLMs to give very varied answers to the same MCQ examination two weeks apart, with consistency rates as low as 61% [15]. To gauge this variability, it would have been beneficial to have each LLM answer the exam multiple times, although this would have introduced a greater risk of data contamination.

## Conclusions

In conclusion, this study demonstrated that a cohort of LLMs performed comparably to high-performing doctors on a demanding undergraduate ophthalmology examination. Consistent with existing literature, the pooled LLM cohort showed no significant difference in overall performance when compared with the doctor cohort. Notably, however, Claude Sonnet 4 achieved a significantly higher score than the doctors, suggesting that individual models may surpass human benchmarks even as the collective performance of LLMs approaches a plateau. The optics sub-analysis, which required application-based reasoning, further indicated that LLMs can handle complex problem-solving beyond factual recall, performing at a level where no significant difference was seen when compared to the doctors. 

These results underscore both the promise and the limitations of LLMs in medical education. While the models exhibit strong potential as supplementary learning tools, their evaluation here was limited to a single MCQ-based exam, which does not fully capture clinical reasoning. Risks such as data contamination and evolving model versions must also be acknowledged. Future research should expand the scope to include larger question banks free from online exposure and focus on analysing the reasoning behind model outputs. Overall, this study highlights the growing utility of LLMs in ophthalmology while reinforcing the ongoing need for critical human oversight. 
